# Overexpression of *OsHAD3*, a Member of HAD Superfamily, Decreases Drought Tolerance of Rice

**DOI:** 10.1186/s12284-023-00647-y

**Published:** 2023-07-19

**Authors:** Xiaofei Zan, Zhanmei Zhou, Jiale Wan, Hao Chen, Jiali Zhu, Haoran Xu, Jia Zhang, Xiaohong Li, Xiaoling Gao, Rongjun Chen, Zhengjian Huang, Zhengjun Xu, Lihua Li

**Affiliations:** 1 State Key Laboratory of Crop Gene Exploration and Utilization in Southwest China, Sichuan Agricultural University, Chengdu, 611130 People’s Republic of China; 2 Rice Research Institute, Sichuan Agricultural University, Chengdu, 611130 People’s Republic of China; 3 Crop Ecophysiology and Cultivation Key Laboratory of Sichuan Province, Chengdu, 611130 People’s Republic of China

**Keywords:** Rice, Drought, Oxidative, Root, Reactive oxygen species

## Abstract

Haloacid dehalogenase-like hydrolase (HAD) superfamily have been shown to get involved in plant growth and abiotic stress response. Although the various functions and regulatory mechanism of HAD superfamily have been well demonstrated, we know little about the function of this family in conferring abiotic stress tolerance to rice. Here, we report *OsHAD3*, a HAD superfamily member, could affect drought tolerance of rice. Under drought stress, overexpression of *OsHAD3* increases the accumulation of reactive oxygen species and malondialdehyde than wild type. *OsHAD3*-overexpression lines decreased but antisense-expression lines increased the roots length under drought stress and the transcription levels of many well-known stress-related genes were also changed in plants with different genotypes. Furthermore, overexpression of *OsHAD3* also decreases the oxidative tolerance. Our results suggest that overexpression of *OsHAD3* could decrease the drought tolerance of rice and provide a new strategy for improving drought tolerance in rice.

**Supplementary Information:**

The online version contains supplementary material available at 10.1186/s12284-023-00647-y.

## Introduction

Abiotic stresses, such as salinization, drought, extreme temperatures, can cause serious damage to agricultural production in worldwide (Akhtar et al. 2021). In recent years, with the intensification of global climate change, the adverse effects of abiotic stress have been further expanded. In order to respond to changes of the external environment, plants can regulate themselves from multiple levels, including molecular, material metabolism and physiological, etc. The stress-related genes, substance metabolism, and reactive oxygen species are the most common response strategies in plants and in-depth exploration of stress-related genes is crucial for enhancing rice stress resistance and increasing crop yield.

The haloacid dehalogenase-like hydrolase (HAD) superfamily includes enzymes that catalyze transfer reactions of carbon or phosphoryl groups on different substrates (Koonin and Tatusov 1994), it is composed mainly of phosphatases (Zhang et al. 2004), ATP enzymes (Collet et al. 1999) and phosphomutase (Collet et al. 1998; May et al. 2011). The core catalytic domain of the HAD superfamily contains a three-layered α/β sandwich, consisting of repetitive β-α units adopting the topology typical of the Rossmanoid class of α/β fold (Pfeiffer et al. 2014).

HAD superfamily consists with many protein family, it’s widely distributed and capable of performing a variety of biological functions (Burroughs et al. 2006), ranging from adjusting the translocation of proteins within mitochondria (Guo et al. 2004) to abscisic acid-responsive transcription (Hugouvieux et al. 2001; Xiong et al. 2002). Polynucleotide kinase phosphatase (PNKP) family, among the members of HAD superfamily, play a role in both DNA and RNA repair by removing 3’-terminal phosphate groups (Jilani et al. 1999). The acid phosphatase family exhibits a lineage-specific expansion of its members in plants and is thought to work as vegetative storage proteins (Gomez and Faurobert 2002). Moreover, the HAD superfamily is also involved in the stress response of plants, and some of these genes are induced by Pi starvation and associated with Pi stress, such as *AtHAD1* and *AtPECP1* (Lee et al. 2022; May et al. 2012). AtSgpp has phosphatase activity and its expression is induced by various stresses. In rice, *OsHAD1* participates in regulating the phosphorylation state of targets associated with the Pi starvation response (Pandey et al. 2017).

Although there are many reports on the involvement of HAD superfamily members in plant growth and stress response, little information about their roles in drought stress response. In the study presented here, we showed that *OsHAD3*, a member of HAD superfamily, could respond to multiple abiotic stresses and overexpression of *OsHAD3* could decrease drought tolerance of rice.

## Materials and Methods

### Plant materials and Growth Condition

Rice (*Oryza sativa* L. subsp*. japonica cv*. Nipponbare) seeds were used as wild type (WT) and materials for genetic transformation. In all experiments, the seeds were soaked with 2% (v/v) NaClO for 30 min, washed them with sterile water and subsequently subjected to imbibition at 37 °C for 3 days in the dark. Then, the germinating seeds were grown in nutrient solution in a grown chamber with a cycle of 16-h light at 28 °C and 8-h dark at 25 °C.

### Construction of Transgenic Plants

Both overexpression and antisense-expression vectors were constructed using the complete coding sequence of *OsHAD3* and the expression vector (D-163 + 1300) was double digested with Hind III and BamH I endonucleases, followed by recombination to construct *OsHAD3*-overexpression and antisense-expression vector. The overexpression primers were F: 5’-tggagaggacagcccaagctt TCATCACGACCTGAAAATCATGG-3’ and R: 5’-gtaccgaattcccggggatcc ACTTCCACGGAACACCCTCC-3’. The antisense-expression primers were F: 5’- tggagaggacagcccaagcttACTTCCACGGAACACCCTCC-3’ and R: 5’-gtaccgaattcccggggatccTCATCACGACCTGAAAATCATGG-3’. In order to get the expression profile of *OsHAD3*, the promoter sequence about 1,500 bp was identified from NCBI (LOC_Os03g16670) and amplified from rice genomic DNA and pCAMBIA1305 vector was double digested with Hind III and Noc I endonucleases, followed by recombination. The GUS primers were F: 5’-gacctgcaggcatgcaagcttAGTTCCCGGCGCCACGTG-3’ and R: 5’-tagaaatttaccctcagatctTCATTCCTCACACGACGTTCAT-3’. The transgenic rice plants were obtained through *Agrobacterium tumefaciens* mediated genetic transformation by infection of wild-type plant callus (Toki et al. 2006) and RT-qPCR was used to detect the transgenic plants.

### RNA Extraction and Quantitative Real Time PCR Analysis

To study the effect of abiotic stress conditions and ABA on the transcript accumulation of *OsHAD3*, three-leaf stage WT (Nipponbare) seedlings were used for stress treatments, including cold (4 °C), hot (42 °C), salt (150 mM NaCl), drought (20% w/v PEG6000) and ABA (50 μM). Leaves were taken at different periods (0 h, 0.5 h, 1 h, 2 h, 4 h, 8 h, 16 h, 24 h and 0 h treatment was the control group) to extract total RNA using Trizol reagent according to the manufacturer's instructions. The reverse transcription was done using a PrimeScript™ RT reagent Kit with a gDNA Eraser kit, and the cDNA was stored at − 20 °C. The extracted cDNA was used as a template to analyze the transcript accumulation of *OsHAD3* under normal and abiotic stress conditions. The initial amount of template cDNA in each amplification reaction was 10 µg. At least three independent biological replicates were performed for each experiment and the rice Ubiquitin gene (Os01g0328400) was used for internal control for qPCR normalization. The 2^−ΔΔCT^ method was used to transform threshold cycle values (Ct) into normalized relative abundance values of mRNA (Livak and Schmittgen 2001). All primer pairs used were listed in Additional file 1: Table S1.

### Histochemical GUS Activity Assay and Subcellular Localization of *OsHAD3*

Different tissues of *OsHAD3p::*GUS transgenic plant were collected and detected following previous method (Jefferson 1989). Different tissues of transgenic rice were placed in a buffer containing 50 mM NaPO_4_ (pH 7.2), 5 mM K_3_Fe (CN)_6_, 5 mM K_4_Fe(CN)_6_, 0.1% (w/w) Triton-100, and 1 mm X-Gluc, and they were incubated overnight at 37 °C and soaked tissues in 70% (v/v) ethanol for 5 min to stop the staining, then, 95% (v/v) ethanol was added and removed chlorophyll completely. Finally, photos were taken with a ZEISS stereo microscope.

The *OsHAD3* full coding sequence (CDS) was removed the stop codon and the D163 + 1300-GFP vector was double digested with Hind III and BamH I endonucleases, followed by recombination to construct OsHAD3-GFP vector, which was transformed into *Agrobacterium tumefaciens* EHA105 and stored at − 80 °C. The GFP primers were F: 5’-tggagaggacagcccaagctt ATGGAGTTCGAAGACCGCTG-3’ and R: 5’-ctcaccatgaccggtggatccGCGGTCACCGATGTCTCGA-3’. Transformed *Agrobacterium* strains were activated and injected into the lower epidermis of tobacco leaves and subsequently cultured for two days under low light. 2 days after tobacco transformation, subcellular localization was observed by confocal laser scanning microscopy (Wang et al. 2021). The 35S: GFP was used as control. All primer pairs used in this study were listed in Additional file 1: Table S1.

### Transgenic Plants Treated with Abiotic Stress

In order to assess the tolerance of transgenic plants to drought stress, the 2-week-old rice plants were transferred into nutrient solution with 20% (w/v) PEG6000 for 10 days, then recovered for 7 days and calculated the survival rates. Besides, the soil experiment was used to further simulate the drought stress in the field. The rice seedlings were cultured in soil for 2 weeks normally, then stopped watering for 7 days until the leaves became curled, followed by a 10-day recovery and calculated the survival rates.

To explore the tolerance of transgenic rice to oxidative stress, the detached leaves of 2-week-old WT and transgenic seedlings were cut down and placed in 1% (v/v) hydrogen peroxide (H_2_O_2_) solution for 3 days to observe the extent of leaf chlorosis. Besides, the 2-day-old seedlings were grown in nutrient solution containing or without 30 μM methyl viologen (MV) for 4 days and observed the growth status.

### Water Loss Rate Measurement

The water loss rates were detected, as previous described with minor modifications (Gao et al. 2020). The leaves of 2-week-old seedlings were sampled at room temperature and the fresh weight was assessed. The detached leaves were placed on dry filter paper at room temperature and weighed at designated time intervals. The water loss rates were calculated as the ratio of the actual weight at different time points to the initial fresh weight. All experiments were performed in at least three independent biological replicates.

### Measurement of the Physiological and Biochemical Indicator

Two-week-old seedlings were transferred to nutrient solution supplemented with 0 or 20% (w/v) PEG6000. 2 days later, the aerial parts of the plants were excised and subjected to the analysis of superoxide dismutase (SOD), peroxidase (POD) activity and content of malondialdehyde (MDA) and soluble sugar as previous describe (Chen et al. 2015) and adjusted. An approximately 0.2 g sample of the aerial tissues was homogenized in 3 mL of 100 mM phosphate buffer (pH 7.8). The homogenate was centrifuged at 10,000×*g* for 10 min at 4 °C and the supernatant was used for the assays. For SOD assays, 0.2 mL of the supernatant was added to 4 mL 100 mM of phosphate buffer (pH 7.8), 0.08 mL 1 mM EDTA-Na_2_, 0.27 mL 130 mM Met, 0.27 mL 750 μM NBT and 0.27 mL 20 μM riboflavin and the absorbance at 560 nm was recorded for 1 min. For POD analysis, 0.2 mL of the supernatant was added to the reaction solution containing 4 mL 100 mM of phosphate buffer (pH 7.0), 2.3 μL of 0.2% (v/v) guaiacol and 2 μL of 30% (v/v) H_2_O_2_ and the absorbance at 470 nm was recorded for 1 min. For MDA content analysis, 0.1 mL of the supernatant was added to 0.4 mL of 0.25% (w/v) thiobarbituric acid (TBA) and the mixture was boiled for 15 min and subsequently cooled on ice for 5 min, the absorbance at both 532 and 600 nm was recorded for 1 min, respectively. Besides, the Nitro Blue Tetrazolium (NBT) and 3’-diaminobenzidine (DAB) were used to detect the content of O^2−^ and H_2_O_2_ in leaves after drought stress, as previous described (Chen et al. 2021). The leaves were incubated in a DAB or NBT solution overnight at 27 °C under light and after staining, the leaves were soaked in 95% ethanol overnight to remove chlorophyll. Meanwhile, the transcript accumulation of ROS-scavenging genes were measured by RT-qPCR. The primer pairs were listed in Additional file 1: Table S1.

### Measurement of the Content of Chlorophyll and Soluble Sugar

The content of chlorophyll was measured as previously described (Gao et al. 2020). The fresh leaves were measured and soaked in 95% ethanol for 48 h in the dark, and then the supernatant was collected to measure the absorbance at 645 nm and 663 nm, respectively. The total chlorophyll content was calculated according to the following formula: Content of Chlorophyll A = 12.7*A663 − 2.69*A645, Content of Chlorophyll B = 22.9*A645 − 4.68*A663, Content of Total chlorophyll (mg/g): (Content [Chlorophyll A] + Content [Chlorophyll B]) * extract volume/sample fresh weight. The anthrone method was used to detect the content of soluble sugar. Approximately 0.1 g of plant samples were crushed and added 15 mL of water and boiled for extraction for 20 min. Strain and dilute the extract to 100 mL with water. Subsequently, 1 mL of the diluted extract was added to 5 mL of anthrone and the mixture was boiled for 10 min and the absorbance was measured by spectrophotometer at 620 nm.

### Bioinformatics and Statistical Analysis

Prior to the study, the sequences of *OsHAD3* gene were downloaded from The Rice Genome Annotation Project Database. The cis-acting elements were found by using the PlantCARE to analyze the promoter sequence of *OsHAD3*. The protein sequences of *OsHAD3* and other species were obtained by NCBI and aligned by using the ESPript website. All experiments were repeated for three times. These data were processed and analyzed by the t-test, with *P* < 0.05 (*) and *P* < 0.01 (**) to be significantly different.

## Results

### Bioinformatics Analyze of OsHAD3

**Fig. 1:**
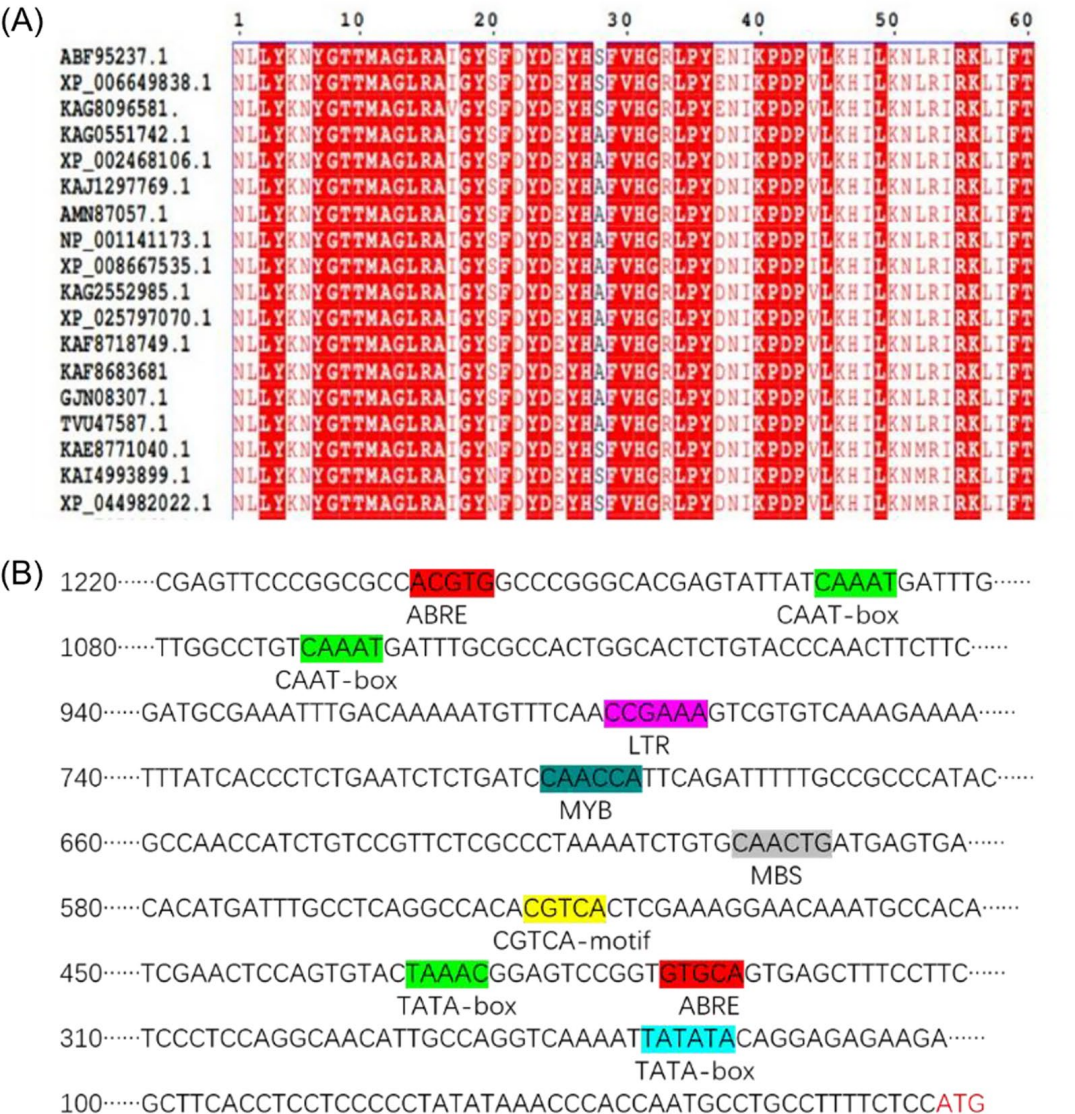
Bioinformatics analysis of *OsHAD3*. **A** Homologous amino acid sequence analysis. **B** Analysis of its promoter sequence

According to its localization on chromosome 3 of rice, we named it as *OsHAD3* (LOC_Os03g16670). It had an open reading frame sequence of 851 bp encoding for 283 amino acids. We compared the protein sequences of *OsHAD3* and its homologous members of some other species, such as Zizania palustris (KAG8096581), Sorghum bicolor (KAG0551742.1, XP002468106.1), Paspalum vaginatum (KAJ1297769.1, AMN87057.1, KAG2552985.1), Zea mays (NP_001141173.1, XP008667535.1, XP_025797070.1), Digitaria exilis (KAF8718749.1, KAF8683681), Eragrostis curvula (TVU47587.1, GJN08307.1) and Hordeum vulgare ( KAE8771040.1, KAI4993899.1, XP_044982022.1) and the result showed a strong conservation between these sequences (Fig. 1A). Meanwhile, the 1.5-kb upstream sequence of translation start site was analyzed by the Plant CARE and found multiple cis-elements associated with the stress response (Fig. 1B), such as ABRE-motif (ABA response element), MBS-motif (MYB binding sites involved in drought induction), CGTCA-motif and TGACG-motif (MeJA-responsiveness), etc.

### *OsHAD3* Could Response to Multiple Abiotic Stress

**Fig. 2:**
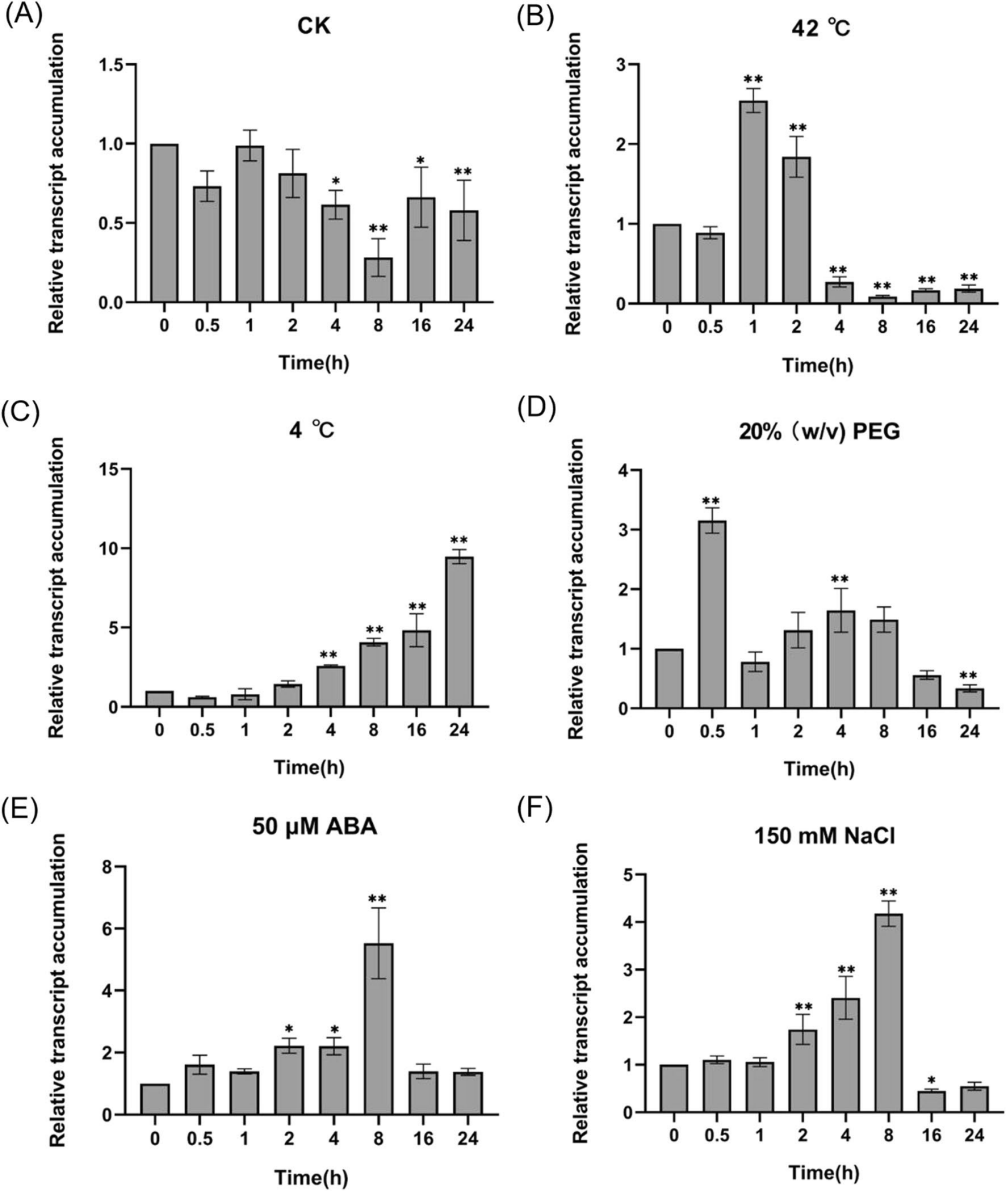
The transcription level of *OsHAD3* in WT at three-leaf stage under abiotic stresses. **A** CK; **B** 42 °C; **C** 4 °C; **D** 20% (w/v) PEG6000; **E** 150 mM NaCl; **F** 50 μM ABA. Data show the mean ± SD of three replicates. Asterisks indicate significant differences between transgenic lines and WT using t-test (*P < 0.05, **P < 0.01)

We performed RT-qPCR analysis to detect the expression pattern of *OsHAD3* by exposing three-leaf stage seedlings to many kinds of abiotic stresses and ABA treatment for a period of 24 h. We found its transcription level was changed to varying degrees, from the minimum 0.08-fold under 42 °C treatment (Fig. 2B) to the maximum 9.4-fold under 4 °C treatment (Fig. 2C). Besides, its highest level appeared at different stress time points when treated with 20% (w/v) PEG6000, 150 mM NaCl and 50 μM ABA treatment, which was 3.2-, 4.1- and 5.5-fold, respectively (Fig. 2D, F). The results showed that *OsHAD3* may be respond to multiple stresses.

### Histochemical GUS Activity and Subcellular Localization of *OsHAD3*

**Fig. 3:**
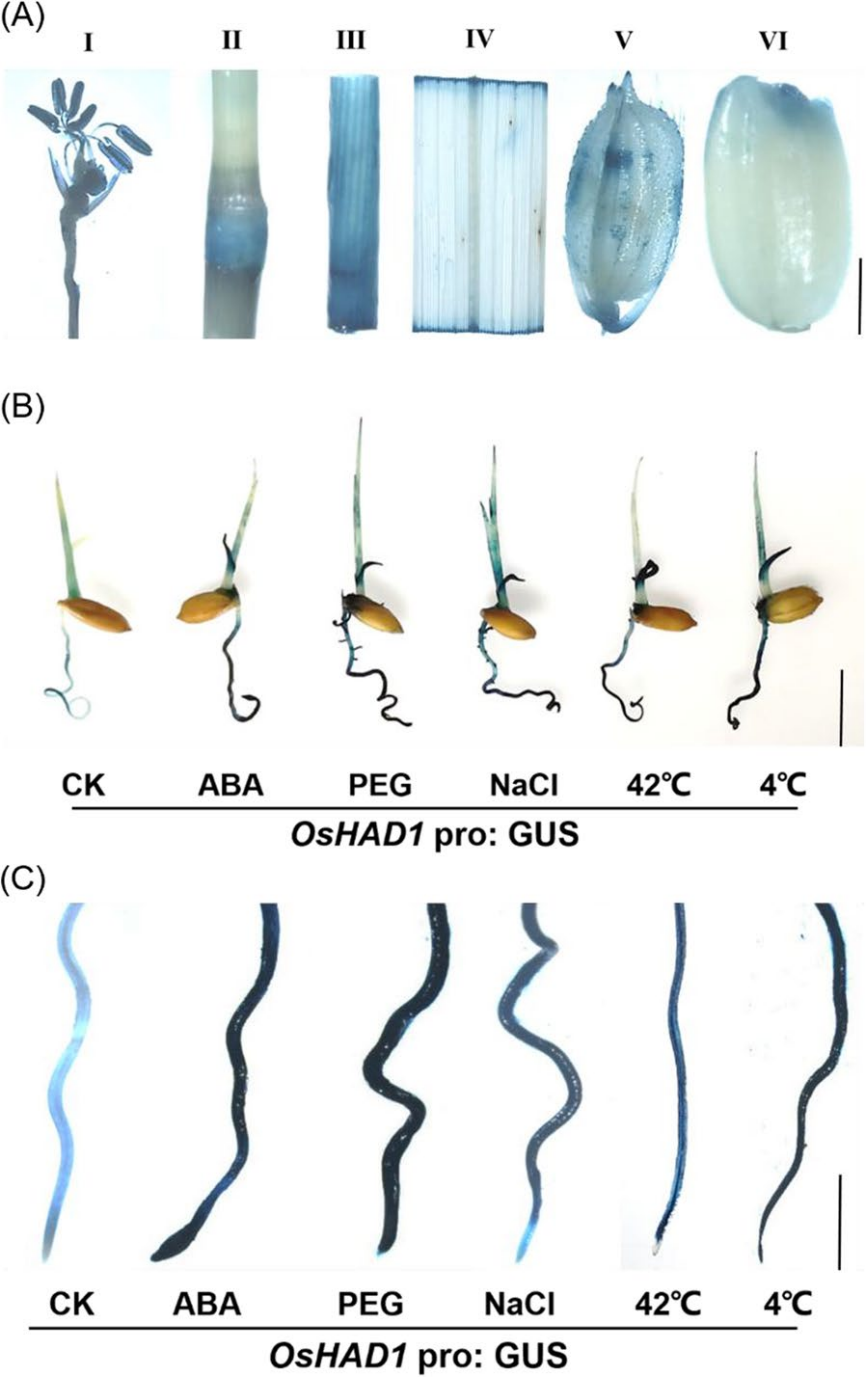
Histochemical GUS activity. **A** GUS staining of different tissues of OsHAD3pro: GUS plants at reproductive stage. (I: anther; II: internode; III: stem; IV: leaf; V: young spikelet hull; VI: seed). Bar, I-IV: 1 cm, V-VI: 0.3 mm. **B** GUS staining of 5-day-old seedlings under abiotic stresses. Bar, 1 cm. **C** GUS staining of 5-day-old roots under abiotic stresses. Bar, 0.5 cm

To analyze the expression pattern of *OsHAD3* in situ, the *OsHAD3p::*GUS transgenic plants were constructed and used for β-glucuronidase (GUS) activity detection. The GUS staining of different tissues, such as anther, internode, young stem, leaf, spikelet hull and seed (Fig. 3A), indicated that *OsHAD3* could constitutively expressed in rice. Furthermore, we investigated the role of *OsHAD3* in stress response, 5-day-old seedlings of *OsHAD3*pro: GUS were treated with 50 µM ABA, 180 mM NaCl, 20% (w/v) PEG6000, 42 °C and 4 °C for 12 h, respectively. Compared with the control group, the results showed a significantly deeper color in the salt, drought and ABA treatments (Fig. 3B). Meanwhile, the GUS staining of roots was deepened to different degrees after the stress treatment, especially after PEG6000 and ABA treatment (Fig. 3C), suggesting furtherly that *OsHAD3* could be induced by multiple stresses.

**Fig. 4:**
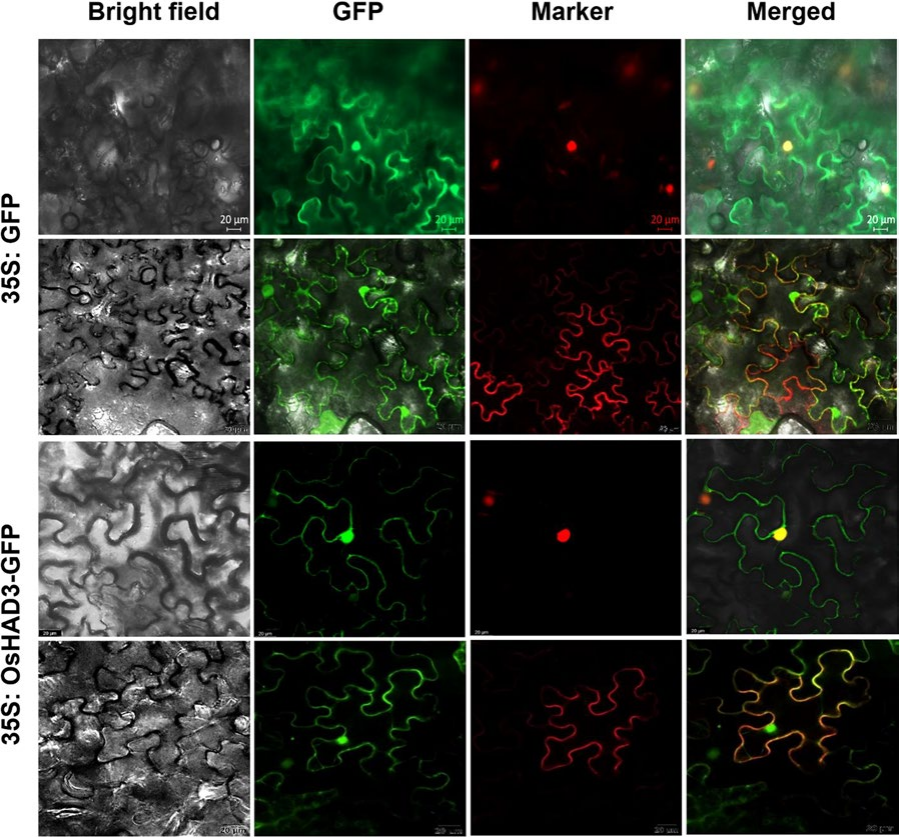
Subcellular localization of *OsHAD3* in tobacco cells. Bar, 20 μm

In order to detect the subcellular localization of *OsHAD3*, the OsHAD3-GFP fusion protein was constructed and tobacco subcellular localization result showed that *OsHAD3* was localized in cell nucleus mainly, partially in cell membrane (Fig. 4).

### Overexpression of *OsHAD3* Reduces, but Antisense-Expression of this Gene Enhances Drought Tolerance in Rice

**Fig. 5:**
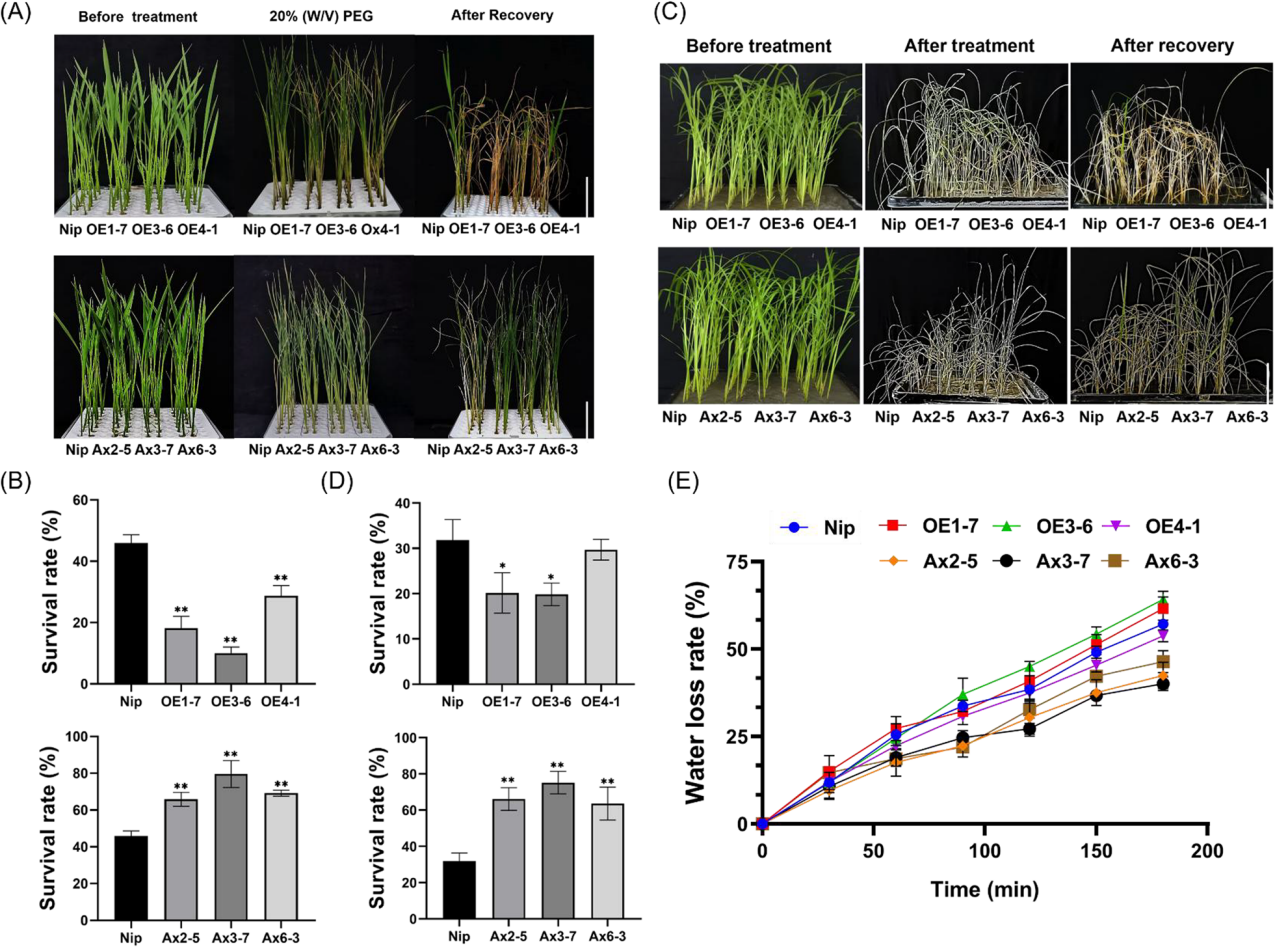
Analyzing drought tolerance of *OsHAD3* transgenic plants. **A** Phenotype of 2-week-old WT and transgenic plants before and after 20% (w/v) PEG6000 treatment for 10 days and recovery for 7 days. **B** Survival rate of seedlings after PEG6000 treatment. **C** Phenotype of 2-week-old seedlings before and after the soil drought experiment for 7 days and recovery for 10 days. **D** Survival rate of soil treatment. **E** Water loss rate of detached leaves of 2-week-old seedlings. Bar, 5 cm. Data show the mean ± SD of three replicates, each replicates contains at least 16 seedlings. Asterisks indicate significant differences between transgenic lines and WT using t-test (*P < 0.05, **P < 0.01)

The transgenic plants were detected by RT-qPCR (Additional file 1: Fig. S1) and to prove that the other members of this family were not affected by the antisense-expression vector, we also examined their transcription level and they were no significant different between WT and antisense-expression lines (Additional file 1: Fig. S2). The seeds of T2 generation from transgenic plants were used for subsequent experiments. Based on the results that *OsHAD3* could be induced by drought (Fig. 2D) and the cis-elements, MYB and MBS-motif, existed in its promoter region (Fig. 1B), we speculated that *OsHAD3* may be involved in plant drought response, therefore we evaluated the performance of 2-week-old plants under drought stress. We found that after a 10-day of 20% (w/v) PEG6000 treatment, the *OsHAD3*-overexpression lines showed more severe chlorosis and wilting but these changes in antisense-expression lines were delayed, compared with that of WT (Fig. 5A) and, followed by a 7-day recovery, the survival rate of WT was lower than antisense-expression lines but higher than *OsHAD3*-overexpression lines (Fig. 5B). Furthermore, we used the soil drought treatment to further simulate the field drought conditions. Compared with WT, after a 7-day withdrawing water followed by 10-day recovery period, the antisense-expression lines retained more green than WT, and *OsHAD3*-overexpression lines were the opposite. A survival rate of approximately 30% of WT was higher than *OsHAD3*-overexpression lines, but lower than antisense-expression lines (Fig. 5C, D). We used the detached leaves of 2-week-old seedlings to preform water loss rate assays and the result showed that compared with WT, the overexpression lines were faster or not significantly different, while the antisense expressing lines reduced the water loss rate (Fig. 5E). Above results confirmed that the *OsHAD3*-overexpression lines were more sensitive but antisense-expression lines were more resistant to drought stress.

### *OsHAD3* Affects Rice Drought Stress Tolerance Through Regulating Root Growth

**Fig. 6:**
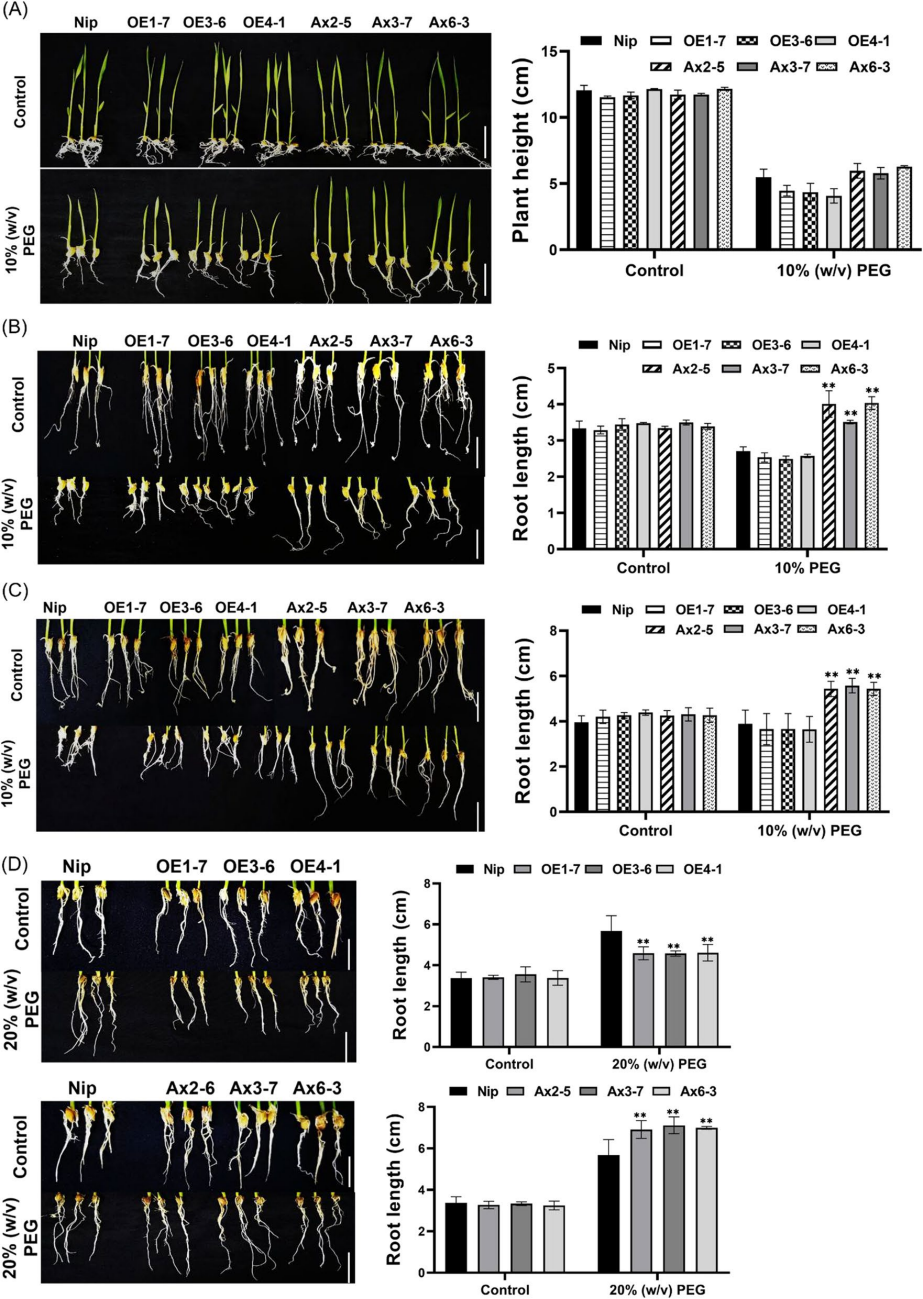
Phenotypes of plants under drought stress. **A** Plant height of 3-day-old seedlings after 7 days of growth under normal or 10% w/v PEG6000 treatment. Bar, control: 3 cm; treatment group: 1 cm. **B**, **C** Root length of 3-day-old plants after 7 and 12 days of growth under normal or 10% w/v PEG6000 treatment. Bar, **B**: 1 cm; **C**: 2 cm. **D** Root length of 2-week-old plants before and after 20% w/v PEG6000 treatment for 10 days. Bar, 2 cm. Data show the mean ± SD of three replicates. Asterisks indicate significant differences between transgenic lines and WT using t-test (*P < 0.05, **P < 0.01)

To investigate the possible mechanism of *OsHAD3* affecting drought tolerance in plants, the 3-day-old WT and transgenic seedlings were grown in nutrient solution containing or without 10% (w/v) PEG6000, and we observed the growth status between them. In control group, no significant difference was observed. After 7 days of treatment, the antisense-expression lines grew better than WT, showing higher plant height, but *OsHAD3*-overexpression lines were slightly suppressed (Fig. 6A). Besides, we found the root growth of the antisense-expression lines was better than WT, while that of overexpression lines were slightly inhibited (Fig. 6B). The difference between the antisense-expression lines and WT was more obvious when the treatment continued to 12 days, showing that the longer roots were observed in antisense-expression lines, while *OsHAD3*-overexpression lines showed no significantly different from WT (Fig. 6C).

To further confirm this phenotype, we increased the concentration of the PEG6000 treatment. 2-week-old seedlings were treated with 20% (w/v) PEG6000 solution for 10 days. We found that after treatment, the root growth of antisense-expression lines grew better than WT, while that of *OsHAD1*-overexpression lines was slightly inhibited (Fig. 6D).

### Overexpression of *OsHAD3* Weakens the Oxidative Stress Tolerance of Rice

**Fig. 7:**
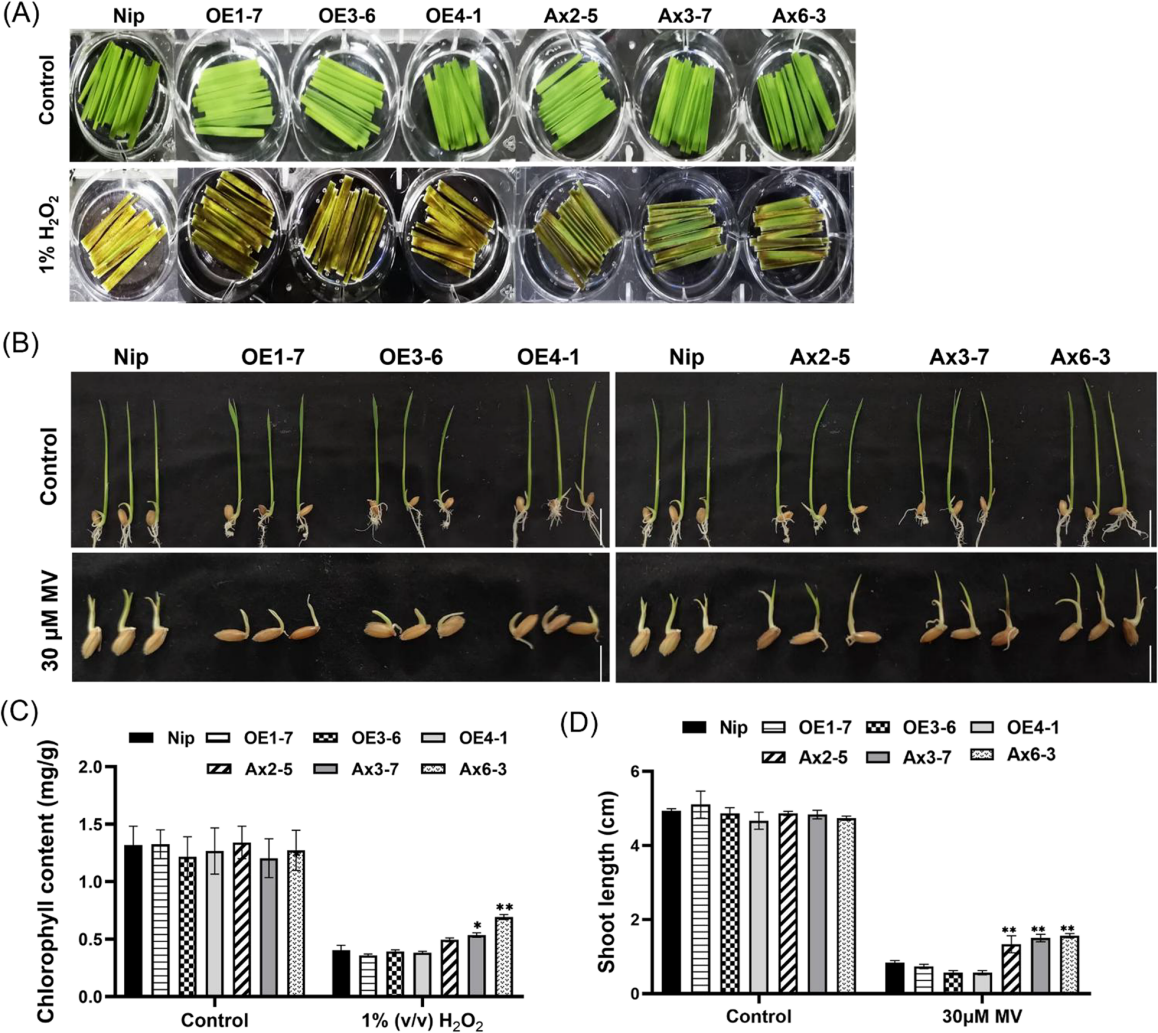
Response of transgenic plants to oxidative stress. **A** Phenotype of 2-week-old detached leaves treated with 1% (v/v) H_2_O_2_ for 3 days. **B** Phenotype of 2-day-old seedlings in nutrient solution with or without 30 μM MV for 4 days. Bar, 1 cm. **C** The chlorophyll content of leaves before and after 3-day H_2_O_2_ treatment. **D** Shoot length of plants in nutrient solution with or without 30 μM MV for 4 days. Data show the mean ± SD of three replicates. Asterisks indicate significant differences between transgenic lines and WT using t-test (*P < 0.05, **P < 0.01)

Overexpression of *OsHAD3* results in decreasing drought stress tolerance of rice indicates that it may have the decreased tolerance to oxidative stress. To confirm this, we used 1% (v/v) hydrogen peroxide (H_2_O_2_) to treat the detached leaves of 2-week-old plants for 3 days. No significant differences in the control group. In the treatment group, the *OsHAD3*-overexpression lines and WT showed severe chlorosis, while the antisense-expression lines had less severe symptom (Fig. 7A). The chlorophyll contents before treatment were no significant difference between plants and after treatment, the chlorophyll contents of antisense-expression lines were higher than WT, but that of overexpression lines were slightly lower or not significantly different from the WT (Fig. 7C). Beside, we transferred 2-day-old seedlings into nutrient solution containing or without 30 μM MV, an oxidative stress inducer in plants. No significant difference in the control group. After treatment for 4 days, the antisense-expression lines had longer shoot length than WT, and overexpression lines were slightly inhibited (Fig. 7B, D). Above results showed that overexpression of *OsHAD3* could decrease the oxidative stress tolerance of rice.

### Overexpression of *OsHAD3* Affects the Level of ROS and Other Stress‑Related Items

**Fig. 8:**
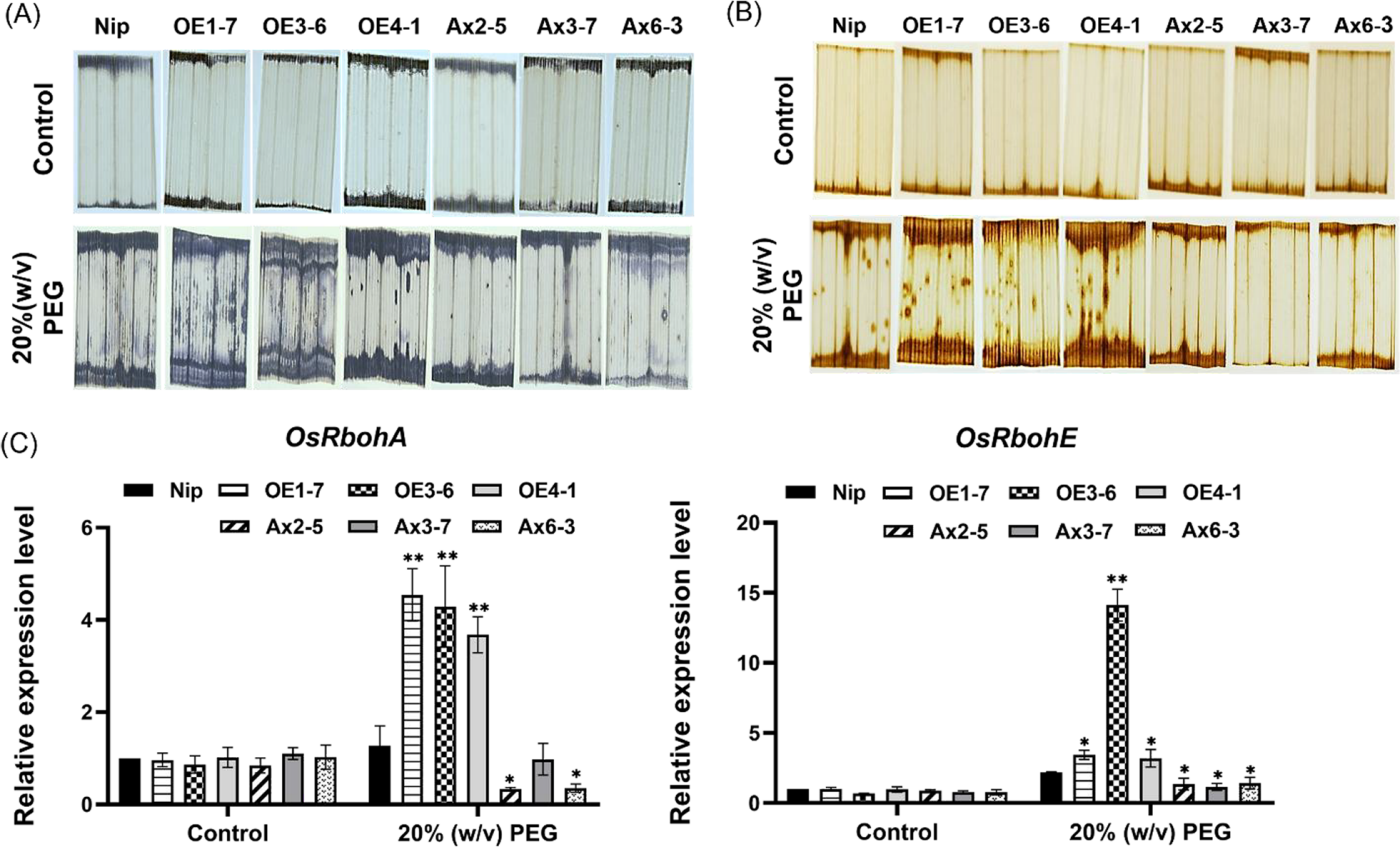
The accumulation of ROS level in leaves of 2-week-old WT and *OsHAD3* transgenic plants before and after 2 days of treatment using 20% (w/v) PEG6000. **A** NBT staining. **B** DAB staining. **C** Transcript accumulation of ROS-production gene, *OsRbohE* and *OsRbohA* before and after treatment. Data show the mean ± SD of three replicates. Asterisks indicate significant differences between transgenic lines and WT using t-test (*P < 0.05, **P < 0.01)

ROS are important signals that regulate the stress tolerance. Here, we used NBT and DAB staining to compare the accumulation of superoxide anion (O^2−^) and hydrogen peroxide (H_2_O_2_) between 2-week-old WT and transgenic plants after drought treatment. No obvious difference in control group. After a 2-day of 20% (w/v) PEG6000 treatment, the results of NBT and DAB staining revealed that leaves of *OsHAD3*-overexpression lines accumulated more O^2−^ and H_2_O_2_ than WT, which were reflected by more severe surface spots and browning surfaces, but these symptoms were all less severe in the antisense expression lines (Fig. 8A, B). The NOX family (Hu et al. 2020) is the key to production of ROS, *OsRbohA* and *OsRbohE* belong to this family and we detected the transcription level of them. We found both of them were increased in overexpression lines but reduced in antisense-expression lines respectively, compared to WT (Fig. 8C), which was consistent with the staining results.

**Fig. 9:**
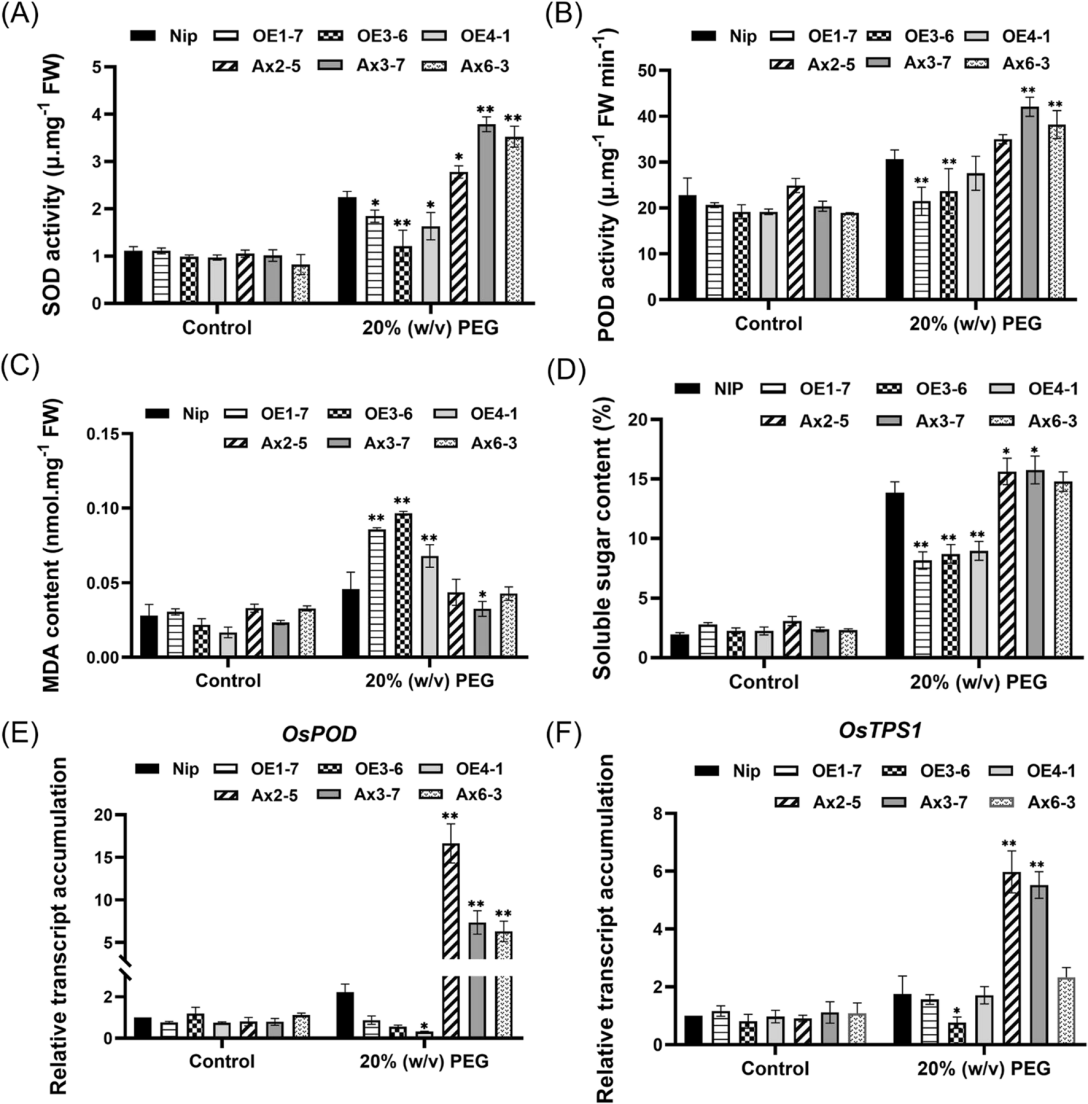
Analysis of SOD and POD activity, MDA and soluble sugar content of WT and transgenic plants under normal and PEG6000-treated conditions. **A** SOD activity. **B** POD activity. **C** MDA content. **D** Soluble sugar content. **E** and **F** Transcript accumulation of *OsPOD*, *OsTPS1* before and after 2-day treatment. Data show the mean ± SD of three replicates. Asterisks indicate significant differences between transgenic lines and WT using t-test (*P < 0.05, **P < 0.01)

In addition, we also detected the activities of ROS-scavenging enzymes (SOD, POD). The results showed that no significant difference between WT and transgenic plants in control group. After 2 days of treatment using 20% (w/v) PEG6000, these enzyme activities were decreased in overexpression lines but increased in antisense-expression lines respectively, compared with WT (Fig. 9A, B). In addition, the content of MDA, compared with WT, was higher in overexpression lines but decreased in antisense-expression lines (Fig. 9C), suggesting the varying degrees of cell membrane damage they suffered. Meanwhile, the content of soluble sugar was increased in antisense-expression lines but decreased in overexpression lines, compared with WT (Fig. 9D). The similar alteration was also observed in the transcription level of *OsPOD* and *OsTPS1*, which are involved in the process of ROS scavenging and trehalose biosynthetic (Fig. 9E, F). Above results indicated that overexpression of *OsHAD3* could decrease POD and SOD activities, thus increasing cell membrane damage and, ultimately, reducing drought stress tolerance.

### Regulation of Transcript Accumulation of Stress-Related Genes in *OsHAD3* Transgenic Plants

**Fig. 10:**
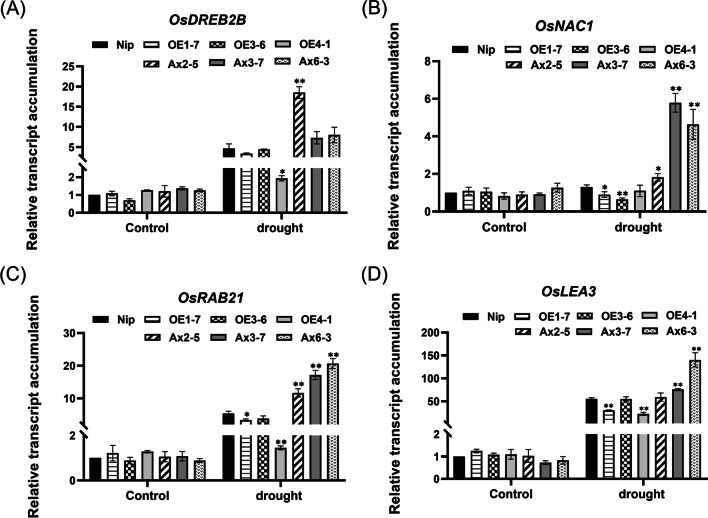
Transcript accumulation of stress-related genes in 2-week-old plants after 8 h of PEG6000 treatment. **A** *OsDREB2B*. **B** *OsNAC1*. **C** *OsLAE3*. **D** *OsRAB21*. Data show the mean ± SD of three replicates. Asterisks indicate significant differences between transgenic lines and WT using t-test (*P < 0.05, **P < 0.01)

To further investigate the possible molecular mechanisms of *OsHAD3* in regulating drought tolerance in plants, we determined the transcript accumulation of some well-known stress-responsive genes. These included *OsDREB2B* and *OsSNAC1*, encoding DREB-type and typical stress-related NAC-type transcription factors (TFs); *OsLEA3* and *OsRAB21*, encoding late embryogenesis abundant (LEA) proteins. After 20% (w/v) PEG6000 treatment for 8 h, compared to WT, the mRNA levels of above genes were both increased in antisense-expression lines, but decreased or not significantly changed in overexpression lines (Fig. 10).

## Discussion

Plants have developed a variety of physiological, cellular, and other ways to survive in deteriorating environments. At the molecular level, a large number of genes were excavated and shown to be involved in the regulation of abiotic stress responses, such as *OsMADS23* and *OsSAPK8*, which are involved in positive regulation of drought and salt tolerance (Zhong et al. 2020), while *OsANN10* and *OsNAC2* act as negative regulators in drought stress response (Gao et al. 2020; Shen et al. 2017).

Members of HAD superfamily has been reported to be involved in the regulation of stress response and plant development processes. In tomato, LePS2 could exert its phosphatase activity, causing anthocyanin accumulation and delayed flowering (Baldwin et al. 2008, 2001). The homologous protein of LePS2, *PvPS2:1*-overexpression in *Arabidopsis* can increase Pi uptake and root growth (Liang et al. 2012). In our research, we identified a member of the HAD family in rice, *OsHAD3*, which encodes a protein localized to both the cell nucleus and membrane. The results of GUS staining and changes in the transcript level of *OsHAD3* in the wild-type under abiotic stress showed that *OsHAD3* could respond to many kinds of abiotic stress.

To further understand the role it plays in abiotic stress response, overexpression and antisense expression lines were constructed and underwent stress treatment. We found that overexpression of *OsHAD3* could decrease drought tolerance of rice, which was confirmed by various stress phenotypes, such as leaf chlorosis and faster water loss rate. The more severe chlorotic phenomenon of detached leaves and shorter shoot length under MV treatment showed that overexpression of *OsHAD3* decrease oxidative stress tolerance of rice. The altered stress tolerance may result from combined changes in multiple aspects, such as morphological, physiological, and molecular level. Roots are the first organ to sense drought stress (Spollen and Sharp 1991), they are very important for plants to cope with complex environments (Meng et al. 2019) and increasing soil water uptake by improving root growth is essential for enhancing stress tolerance (Yue et al. 2006). Overexpression of *OsZFP350* could improve roots development and resistance to abiotic stress (Kang et al. 2019). *AtHDG11* improve drought and salt tolerance in transgenic cotton by forming longer and denser root system (Yu et al. 2016). Drought activates *YUCCA7* (*YUC7*) gene, and under its activation, *yuc7-1D* mutant showed significantly increased lateral root numbers and enhanced drought tolerance in *Arabidopsis* (Lee et al. 2012). In this study, we found that antisense-expression lines formed longer root under drought treatment, which was similar to these previous results and *OsHAD3*-overexpression lines had shorter roots. The different root lengths of transgenic plants may be one of reasons of altered drought resistance in plants.

Reactive oxygen species (ROS), as one of the normal by-products in the process of aerobic metabolism, act as secondary messenger to transport signaling during severe stresses (Nadarajah 2020). In normal condition, the balance between production and scavenging is used to keep homeostasis and low levels of ROS in cell (Mittler et al. 2004). But when facing stresses, the amounts of ROS could be excessive and toxic to cells (Hussain et al. 2018; Sewelam et al. 2016). Improving the antioxidant activity to reduce the over-production of ROS is the most useful way to enhance the stress tolerance in plants (Panda et al. 2021), such as enhancing the activity of the POD and SOD (Gill and Tuteja 2010). Overexpression of *OsMLP423* or *OsSCL30* both reduced the damage caused by ROS through enhancing the activities of SOD and POD (Zhou et al. 2022; Zhang et al. 2022). Our result showed that overexpression of *OsHAD3* could increase the over-accumulation of ROS but antisense-expression lines were opposite under drought stress condition, which could be further confirmed by the staining results of DAB and NBT and transcription level of genes, *OsRbohA* and *OsRbohE*. Moreover, we found the activity of POD and SOD in overexpression lines were both lower than WT and antisense-expression lines were opposite. MDA, which acts as an indicator of the degree of cell membrane damage and lipid peroxidation initiated by ROS (Zhang et al. 2021). The result that the MDA content of transgenic plants, which was consistent with stress phenotype, indicating that overexpression of *OsHAD3* increased lipid peroxidation in plants. Thus, we speculated that overexpression of *OsHAD3* could increase the accumulation of ROS and reduce the scavenging system activity to aggravate damage caused by drought stress induced excess ROS, further decreasing drought tolerance of rice.

Another important change of *OsHAD3* transgenic plant is the accumulation of osmolytes. In response to stress conditions, plants accumulate some materials, such as proline, soluble sugars and proteins to maintain turgor pressure and reduce the cell damage from ROS (Wang et al. 2003). As one of the osmolytes, the content of soluble sugar increases under water deficit and it could play a role in drought resistance in rice (Panda et al. 2021). As one of the soluble sugar components, trehalose plays an important role in abiotic stress tolerance and *OsTPS1* is used for the synthesis of trehalose. Overexpression of *OsTPS1* can improve trehalose content and enhance drought stress tolerance (Li et al. 2011). In our results, transcription level of *OsTPS1* decreased and increased in overexpressed and antisense lines, respectively, which was consistent with the variation of soluble sugar content. Late embryogenesis abundant (LEA) proteins are highly hydrophilic and could maintain water, and they are accumulated in response to drought, salinity and protect cellular structure by adjusting osmotic pressures (Chakrabortee et al. 2007). In our study, the LEA-encoding genes, the transcription level of *OsLEA3* and *OsRAB21* were down- and up-regulated in *OsHAD3*-overexpression and antisense-expression plants after PEG6000 treatment, respectively. Therefore, we speculated that overexpression of *OsHAD3* may affect transcription levels of related synthetic genes, such as *OsTPS1*, *OsLEA3* and *OsRAB21* and thus lead to change content of osmotic substances, such as soluble sugar, further decreasing the drought tolerance.

Drought stress causes plant water deficit and under this situation, the expression change of gene (be up- or down-regulated) take place (Farooq et al. 2009) and these genes encode a variety of proteins involved in physiological and biochemical processes and, in addition to the LEA proteins, multiple regulatory factors are also included, such as transcription factors (Almeida et al. 2016). Many transcription factors as critical roles in regulating stress response in plants include *OsDREB2B* and *OsSNAC1*, which improve drought tolerance when overexpressed (Chen et al. 2008; Liu et al. 2014) and in our result, they were both increased transcription level in antisense-expression lines but reduced in overexpression lines, compared with WT. Meanwhile, it has been shown that overexpression of *OsDREB2B*, *CYP735A* and *OsDREB1F* are able to increase rice root morphological adaptation under drought stress (Kim et al. 2020). The similar result was existed in our research that better developed roots as well as higher expression level of *OsDREB2B* were observed in antisense expression lines. These imply, therefore, that overexpression of *OsHAD3* could decrease drought tolerance of rice by affecting the transcript accumulation of these stress-responsive genes.

## Conclusion

In this study, we explored the bio-function of *OsHAD3* in abiotic stress response. Our results show that *OsHAD3*-overexpression plants are more sensitive to drought stress, but the antisense-expression lines are more tolerant. These results provide a reference for further insights into the function of *OsHAD3* and developing potential candidate genes for drought-resistant transgenic rice varieties.

## Supplementary Information

**Additional file 1. Figure S1** Detection of transcript level of OsHAD3 in transgenic plants. **Figure S2** Detection of transcript levels of other family members in WT and transgenic plants. **Table S1** Primers used in this study.

## Data Availability

All data generated or analyzed during this study are included in this published article (and its Additional file 1).

## Abbreviations

HAD: Haloacid dehalogenase-like hydrolase
GFP: Green fluorescent protein
GUS: β-Glucuronidase
NBT: Nitrotetrazolium blue chloride
DAB: 3′-Diaminobenzidine
POD: Peroxidase
SOD: Superoxide dismutase
MDA: Malondialdehyde
