# Supplementary material for: Overexpression of OsHAD3, a Member of HAD Superfamily, Decreases Drought Tolerance of Rice
Source: Rice (N Y). 2023 Jul 19;16:31. doi: 10.1186/s12284-023-00647-y (PMC10356738; doi:10.1186/s12284-023-00647-y)
Supplement: Supplementary file 1 — Additional file 1. Figure S1 Detection of transcript level of OsHAD3 in transgenic plants. Figure S2 Detection of transcript levels of other family members in WT and transgenic plants. Table S1 Primers used in this study. [file 12284_2023_647_MOESM1_ESM.docx]

Additional file 1

Overexpression of *OsHAD3*, a member of HAD superfamily, decreases drought tolerance of rice.

Xiaofei Zan ^a 1^, Zhanmei Zhou ^a 1^, Jiale Wan ^a^, Hao Chen ^a^, Jiali Zhu ^a^, Haoran Xu ^a^, Jia Zhang ^a^, Xiaohong Li ^a^, xiaoling Gao ^abc^, Rongjun Chen ^abc^, Zhengjian Huang ^abc^, Zhengjun Xu ^abc^ ^*^, Lihua Li ^abc^ ^*^

^a^ Rice Institute of Sichuan Agricultural University, Chengdu, 611130, PR China

^b^ State Key Laboratory of Crop Gene Exploration and Utilization in Southwest China, Sichuan Agricultural University, Chengdu, 611130, PR China

^c^ Crop Ecophysiology and Cultivation Key Laboratory of Sichuan Province, Chengdu, 611130, PR China

^1^These authors contributed equally to this work.

*** Correspondence** **Professor:**

Zhengjun Xu: [mywildrice@aliyun.com](mailto:mywildrice@aliyun.com) Lihua Li: lilihua1976@tom.com.

# Supplementary Figures and Tables

## Supplementary Figures

A B


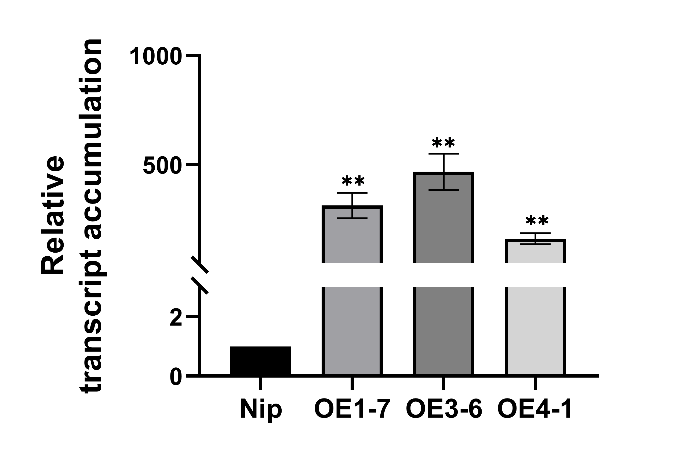

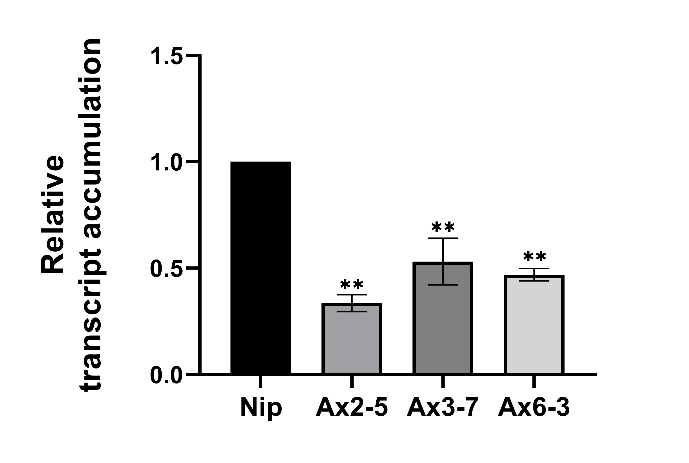


**Supplementary Figure S1. The transcript level of *OsHAD3* in transgenic plants.** (A) The transcript level of *OsHAD3* in overexpression lines. (B) The transcript level of *OsHAD3* in antisense-expression lines. Values are mean ± SE (n = 3, three biological replicates). Asterisks indicate significant differences between transgenic lines and WT using t-test (* *P* < 0.05, ** *P* < 0.01)


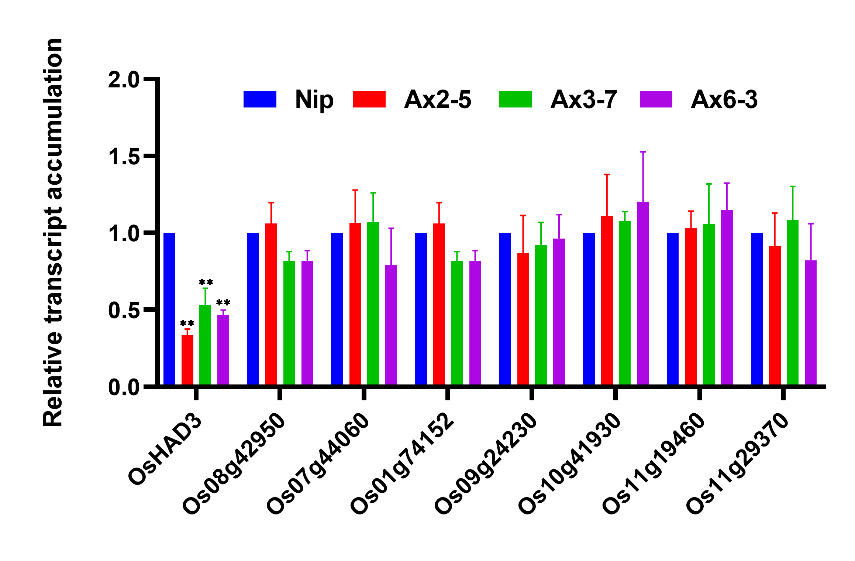


**Supplementary Figure S2. The transcript level of other family members in WT and transgenic plants.** Compared with *OsHAD3*, the transcript level of other members was no significant different between WT and antisense-expression lines. Values are mean ± SE (n = 3, three biological replicates). Asterisks indicate significant differences between transgenic lines and WT using t-test (* *P* < 0.05, ** *P* < 0.01)

## Supplementary Tables

Supplementary Table S1 Primers used in this study

| Primers for generating DNA vectors | |
| --- | --- |
| Primer | Sequences |
| *OsHAD1*-OE-F | 5’-tggagaggacagcccaagcttTCATCACGACCTGAAAATCATGG-3’ |
| *OsHAD1*-OE-R | 5’-gtaccgaattcccggggatccACTTCCACGGAACACCCTCC-3’ |
| *OsHAD1*-Ax-F | 5’-tggagaggacagcccaagcttACTTCCACGGAACACCCTCC-3’ |
| *OsHAD1*-Ax-R | 5’-gtaccgaattcccggggatccTCATCACGACCTGAAAATCATGG-3’ |
| *OsHAD1*-GFP-F | 5’-tggagaggacagcccaagcttATGGAGTTCGAAGACCGCTG-3’ |
| *OsHAD1*-GFP-R | 5’-ctcaccatgaccggtggatccGCGGTCACCGATGTCTCGA-3’ |
| *OsHAD1*-GUS-F | 5’-gacctgcaggcatgcaagcttAGTTCCCGGCGCCACGTG-3’ |
| *OsHAD1-*GUS-R | 5’-tagaaatttaccctcagatctTCATTCCTCACACGACGTTCAT-3’ |
| Primers for RT-qPCR | |
| *OsHAD1-*OE-F | 5’-ATTCGACTGCCTGCTGTTTG-3’ |
| *OsHAD1-*OE-R | 5’-ACAGCAGATTGCCAAGGTTC-3’ |
| *OsHAD1-*Ax-F | 5’-CAGCAATGTTGGAACGGGTC-3’ |
| *OsHAD1-*Ax-R | 5’-TCAGAGCTTCAGTCTAAACACGA-3’ |
| *OsPOD*-F | 5’-AACGCAACCACCAAGCCG-3’ |
| *OsPOD*-R | 5’-CCTCGATCATGCCCATCTTGA-3’ |
| *OsRbohA-F* | 5’-GAGCGCGTCTGCCAATAAAC-3’ |
| *OsRbohA-R* | 5’-TCAATGTAGCCGAGCCCTTC-3’ |
| *OsRbohE*-F | 5’-TCCAATCAGTGTAGCGTGGC-3’ |
| *OsRbohE*-R | 5’-TTTGAATGACCGGCGCTTTG-3’ |
| *OsLEA3*-F | 5’-TGAAGAGCACGGTGGTCGG-3’ |
| *OsLEA3*-R | 5’-GGCAGAGGTGTCCTTGTTGG-3’ |
| *OsSNAC1*-F | 5’-GGGTCAAGACTGATTGGATCAT-3’ |
| *OsSNAC1*-R | 5’-CATCTTCTCCCACTCGTTCTTC-3’ |
| *OsDREB2B*-F | 5’-GTGGAGGCGAGGAAAGTACTGGA-3’ |
| *OsDREB2B*-R | 5’-CCTGTGGATCAAGCTCCTGC-3’ |
| *OsRAB21*-F | 5’-CACACCACAGCAAGAGCTAAGTG-3’ |
| *OsRAB21*-R | 5’-TGGTGCTCCATCCTGCTTAAG-3’ |
| *OsTPS1-F* | 5’-CCGCTACCTGCTTAACTCGT-3’ |
| *OsTPS1-R* | 5’-ACTGGAACCCATTGTGAGCC-3’ |
| *Os08g42950-F* | 5’-ACATCAGCAATGTCCCTGGC-3’ |
| *Os08g42950-R* | 5’-TTGATGTCGTGGTGCAGTGA-3’ |
| *Os07g44060-F* | 5’-CGGATTCCACACGGTGATCG-3’ |
| *Os07g44060-R* | 5’-CGCCTCCTTGATGTTGTGGA-3’ |
| *Os01g74152-F* | 5’-CAAAGTCGCCAATCCTGTGC-3’ |
| *Os01g74152-R* | 5’-CGGATTGATGGAGGCAACCT -3’ |
| *Os09g24230-F* | 5’-CCAACGGCATCCCAGCTTC-3’ |
| *Os09g24230-R* | 5’-CTGAGTCGCACAAGGTCCC-3’ |
| *Os10g41930-F* | 5’-GCTGTCAAAGCTTGCTCCAC-3’ |
| *Os10g41930-R* | 5’-TGTTGAAACCAACCTGCTGC-3’ |
| *Os11g19460-F* | 5’-CAAGCATCCAACAGCATGGTC-3’ |
| *Os11g19460-R* | 5’-GCATGCTTTCAAACCTTCCA-3’ |
| *Os11g29370-F* | 5’-GCATGCTTTCAAACCTTCCA-3’ |
| *Os11g29370-R* | 5’-CCATGCTCAGGAGTTGTCCA-3’ |
| *UBQ5*-F | 5’-ACCACTTCGACCGCCACTACT-3’ |
| *UBQ5*-R | 5’-ACGCCTAAGCCTGCTGGTT-3’ |
